# Enabling Efficient Genetic Manipulations in a Rare Actinomycete *Pseudonocardia alni* Shahu

**DOI:** 10.3389/fmicb.2022.848964

**Published:** 2022-03-03

**Authors:** Jie Li, Baiyang Wang, Qing Yang, Han Si, Yuting Zhao, Yanli Zheng, Wenfang Peng

**Affiliations:** ^1^State Key Laboratory of Biocatalysis and Enzyme Engineering, Hubei Engineering Research Center for Bio-enzyme Catalysis, Environmental Microbial Technology Center of Hubei Province, School of Life Sciences, Hubei University, Wuhan, China; ^2^College of Life Science and Technology, Wuhan Polytechnic University, Wuhan, China; ^3^Department of Microbiology, College of Life Sciences, Wuhan University, Wuhan, China

**Keywords:** *Pseudonocardia*, genetic manipulation, conjugal transfer, gene integration, gene deletion

## Abstract

*Pseudonocardia* species are emerging as important microorganisms of global concern with unique and increasingly significant ecological roles and represent a prominent source of bioactive natural products, but genetic engineering of these organisms for biotechnological applications is greatly hindered due to the limitation of efficient genetic manipulation tools. In this regard, we report here the establishment of an efficient genetic manipulation system for a newly isolated strain, *Pseudonocardia alni* Shahu, based on plasmid conjugal transfer from *Escherichia coli* to *Pseudonocardia*. Conjugants were yielded upon determining the optimal ratio between the donor and recipient cells, and designed genome modifications were efficiently accomplished, including exogenous gene integration based on an integrative plasmid and chromosomal stretch removal by homologous recombination using a suicidal non-replicating vector. Collectively, this work has made the *P. alni* Shahu accessible for genetic engineering, and provided an important reference for developing genetic manipulation methods in other rare actinomycetes.

## Introduction

Bacteria in the rare genus of *Pseudonocardia* belong to *Actinomycetes* that are represented by Gram-positive bacteria with high G + C DNA content (Tiwari and Gupta, 2012). With recent rapid developments in genome sequencing, more and more *Pseudonocardia* genomic data have been accumulated in the database. Many *Pseudonocardia*-specific gene clusters are now available, and their functions, concerning, for example, synthesis of many natural bioactive products (Miyamoto et al., 2014; Caffrey et al., 2016; Won et al., 2017; Geddis et al., 2018; Fang et al., 2019; Strecker et al., 2019; Subramani and Sipkema, 2019), biodegradation of large amounts of industrially discharged environmental pollutants (Grostern et al., 2012; Sales et al., 2013; Inoue et al., 2016; Yamamoto et al., 2018; Qi et al., 2019), defense against invading genetic elements (Doron et al., 2018), have been and will continue to be deduced or demonstrated, showing great application potentials in human medicine, animal health, and crop protection, etc. Indeed, current applications of *Pseudonocardia* have been summarized very recently (Riahi et al., 2021). These beneficial potentials have attracted a great deal of interest in the *Pseudonocardia* genus. However, the generally poor efficiency or perhaps lack of genetic manipulation tools for *Pseudonocardia* has largely impeded deeper research and further application of this genus.

Introduction of DNAs into actinomycetes is generally difficult, possibly owing to the strong cell wall of such bacteria. Although strategies including protoplast transformation and electroporation of plasmids have been successfully used to introduce DNAs into some *Streptomyces* species, they are either of low efficiency or not adaptable for many of the less easy-to-handle non-model actinomycetes. Fortunately, easy and efficient DNA introduction from *Echerichia coli* to actinomycetes can be achieved *via* conjugal transfer. This method represents probably the most straightforward strategy for genetic manipulation in actinomycetes.

Conjugal transfer of different types of vectors, including replicative plasmids, integrative plasmids, and suicidal non-replicating vectors, has been applied in various actinomycetes. The replicative plasmids are useful for overproduction of heterologous proteins, but their host range is usually restricted to native strains and permanent selective pressure is required for their stable maintenance in the hosts. Advantageously, the other two types of vectors, integrative plasmids and suicidal non-replicating vectors, can introduce stable genomic alterations in many actinomycetes. Integrative plasmids contain genes encoding attachment/integration (*att*/*int*) functions, therein being capable of incorporating DNAs into chromosomes of a range of actinomycete species. Mechanistically, integrases derived from bacteriophages catalyze site-specific recombination between attachment sites, respectively, located on the genomes of phage (*attP*) and bacteria (*attB*), forming *attL* and *attR* hybrid sites (Combes et al., 2002). Currently, the suicidal non-replicating vectors have been extensively used to generate stable DNA inserts into any neutral genomic region, or deletion of genes of interest, *via* homologous recombination.

Here we report a rare actinomycete strain, *Pseudonocardia alni* Shahu, recently isolated from the sediment of a lake in the Shahu Park, Wuhan, China. In order to manipulate *P. alni* Shahu, we established an efficient conjugal transfer system, and *via* which, conjugal transfer of the pSET152 plasmid (Flett et al., 1997) and its derivatives from *E. coli* cells to *P. alni* spores were successfully achieved. Moreover, upon conjugation, the pSET152 derivatives have mediated the accomplishment of stable genomic alterations, including integration of an exogenous *gfp* gene into the chromosome and deletion of the *pgl* genes encoding key elements of a phage growth limitation antiviral defense system (Goldfarb et al., 2015). Collectively, these findings have allowed us to conduct genetic analyses of gene or system functions in *P. alni* Shahu, which, potentially, may serve as an important resource for both theoretical research and practical application in the future.

## Materials and Methods

### Bacterial Strains and Culture Conditions

*Pseudonocardia alni* Shahu and derivatives constructed in this work, and *E. coli* strains, were listed in Supplementary Table S1. *Pseudonocardia alni* Shahu cells were grown at 30°C in an ISP4 medium (10 g/L soluble starch, 1 g/L K_2_HPO_4_, 1 g/L MgSO_4_·7H2O, 1 g/L NaCl, 2 g/L (NH_4_)_2_SO_4_, 2 g/L CaCO_3_, 0.001 g/L FeSO_4_·7H_2_O, 0.001 g/L MnCl_2_·4H_2_O, and 0.001 g/L ZnSO_4_·7H_2_O, and pH 7.0–7.4) or on ISP4 agar plates (15 g/L agar in ISP4), while *E. coli* strains at 37°C in Luria broth or on Luria agar plates. When required, the following antibiotics were used: 50 mg/L of apramycin, 25 mg/L of chloramphenicol, and 50 mg/L of kanamycin.

### Construction of Plasmids

All the conjugation plasmids were constructed based on the pSET152 vector and listed in Supplementary Table S1. The site-specific integration plasmid pInt-green fluorescent protein (GFP) was generated by inserting a GFP-expression cassette into pSET152 at BamH I and Not I sites (Supplementary Figure S1). The GFP-expression DNA fragment was synthesized from GenScript (Nanjing, China). To construct the non-replicating suicidal plasmid, we first amplified three DNA fragments by PCR with each containing either the *E. coli ori* (primer set P1 + P2), or the *aac(3)IV* gene encoding the apramycin-resistance marker (P3 + P4), or the elements mediating plasmid transfer, i.e., *traJ* and *oriT* of RK2 (P5 + P6), using pSET152 as a template. These fragments were then ligated together using T4 ligase to generate a base vector, pTSF, for plasmid transfer. Subsequently, the upstream (Up, P7 + P8) and downstream (Down, P9 + P10) sequences of the target *pgl* genes were amplified from the genomic DNA of *P. alni* Shahu by PCR and inserted into pTSF as homologous recombination arms using EcoR I and Sal I, Hind III, and Pvu II sites, respectively, giving pKO-pgl (Supplementary Figure S1). All oligonucleotides were synthesized from GenScript (Nanjing, China) and listed in Supplementary Table S2. Restriction enzymes and T4 ligase were purchased from New England Biolabs (Beijing) Led (Beijing, China).

### Intergeneric Conjugation With *E. coli* and Screening of Recombinants

*Pseudonocardia alni* Shahu spores and the methylation-deficient *E. coli* strain ET12567 containing pUZ8002 were used as the recipient and the donor, respectively, throughout this study. A culture of the donor cells containing a transfer plasmid was grown to an OD_600_ of 0.4 in the presence of 50 mg/L apramycin, 50 mg/L kanamycin, and 25 mg/L chloramphenicol. The cells were then washed twice with an equal volume of LB to remove the antibiotics and finally resuspended in 0.1 volume of LB. The *P. alni* Shahu spores were resuspended with 0.5 ml of 2 × YT broth (10 g/L yeast extract, 16 g/L trptone, and 5 g/L NaCl). Recipient spores and donor cells were mixed and spread on ISP4 agar plates. After incubation at 30°C for 12–13 h, the plates were overlaid with 1 ml of distilled water containing 0.75 mg nalidixic acid and 1.25 mg apramycin, and put at 30°C until colonies were observed. Recombinant candidates were screen by colony PCR using primers listed in Supplementary Table S2. The PCR products were analyzed by agarose gel electrophoresis and confirmed by Sanger sequencing (GneScript, Nanjing, China).

### FACS Analysis

The protocol used for FACS analysis was as previously described with slight modifications (Zheng et al., 2019). The *P. alni* Shahu cells were washed with phosphate buffered saline (PBS) twice and then resuspended into PBS to a concentration of 10^7^ cells/ml. Afterwards, the cells were analyzed *via* flow cytometry using Beckman CytoFLEX FCM (Beckman Coulter, Inc., United States) with PBS as the sheath fluid. A 488 nm laser was used as an FITC channel to detect the cell fluorescence of GFP.

## Results

### Identification and Characterization of *P. alni* Shahu

**Figure 1:**
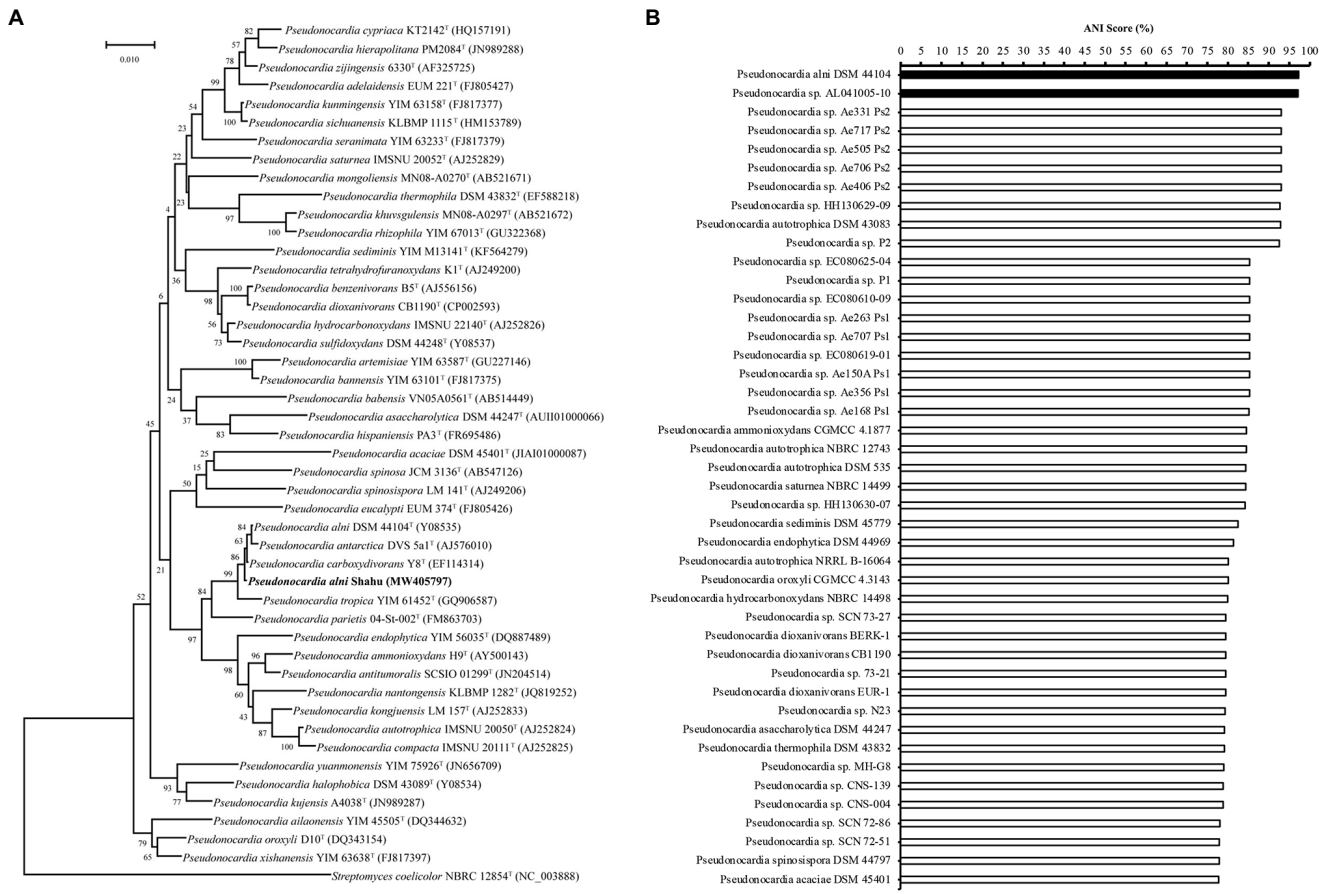
Identification of *Pseudonocardia alni* Shahu. **(A)** Neighbor-joining phylogenetic tree based on 16S rDNA sequences showing the phylogenetic relationships of *P. anli* Shahu and the selected *Pseudonocardia* species. *Streptomyces coelicolor* NBRC 12854^T^ represents an outgroup reference. **(B)** Taxonomic analysis of *P. anli* Shahu with genomes of *Pseudonocardia* available in the NCBI Genome database. Pairwise Average Nucleotide Identity (ANI) score was estimated, and ANI score over 95% indicated with filled bars.

In order to identify the isolate, based on the complete genome sequence that has been deposited into NCBI with the BioProject accession number PRJNA563468, the 1,540-bp 16S rDNA gene sequence (GenBank accession number MW405797, Supplementary Table S3) was phylogenetically characterized *via* BLAST (NCBI) search. In the constructed phylogenetic tree, the isolate falls into the cluster including members of *P. alni*, *Pseudonocardia antarctica*, and *Pseudonocardia carboxydivorans* with a reliability of 86% (Figure 1A), suggesting that it belongs to the genus *Pseudonocardia*. To further detail the species of this isolate, its genome sequence was then subjected to a taxonomic analysis with genomes of *Pseudonocardia* available in the NCBI Genome database to estimate pairwise Average Nucleotide Identity (ANI) score using the FastANI method (https://github.com/ParBLiSS/FastANI; Jain et al., 2018). The result showed that the genome of the isolate pairing with that of *P. alni* DSM44104^T^ or that of *Pseudonocardia* sp. AL041005-10 had ANI values ≥95% (Figure 1B), a criterion determining the same species in an existing genus (Jain et al., 2018). Taken together, the isolate was identified to be *P. alni* and assigned the strain name Shahu.

In order to determine a proper antibiotic resistance marker for *P. alni* Shahu, we investigated the resistance profile of this bacterium to common selective antibiotics, including apramycin (50 mg/L), kanamycin (50 mg/L), chloramphenicol (25 mg/L), tetracycline (50 mg/L), streptomycin (50 mg/L), and ampicillin (100 mg/L). About 10^6^ pores were plated onto ISP4 agar containing one of the above antibiotics. At the tested concentrations, *P. alni* Shahu was sensitive to apramycin, chloramphenicol, and tetracycline, while fully resistant to kanamycin, streptomycin, and ampicillin.

### Accomplishment of Conjugal Transfer Upon Determining the Optimal Donor-to-Recipient Ratio

Conjugal transfer represents one of the most straightforward and efficient methods for introducing foreign DNAs into rare *Antinomycetes*. We initially attempted conjugation between *E. coli* ET12567 (pUZ8002, pSET152) and *P. alni* Shahu following a standard conjugation protocol (Rebets et al., 2017), but failed in obtaining any exconjugant on the plate containing apramycin. This suggested that a modified protocol that specifically works for this strain is required to be established.

Previous studies have reported that an appropriate concentration of Mg^2+^ in the medium, or a suitable heat-shock treatment of the cells, or an optimal ratio of donor-to-recipient could give improved frequency of conjugation (Song et al., 2019; Zhang et al., 2019). We thus individually assessed each of such factors to attain successful conjugation. While, still, no exconjugant could be yielded, neither in the assay examining the various Mg^2+^ concentrations (0, 5, 10, 15, 30, and 50 mM), nor in that testing the heat-shocks with a temperature range from 37 to 65°C (data not shown). Fortunately, when we attempted changing the ratios of donor cells to recipient spores, we saw that different numbers of exconjugants appeared on the selective plates. The number of recipient cells played a more decisive role than that of the donor cells in the efficiency of conjugal transfer between *E. coli* and *P. alni*. When donor and recipient cells were 1 × 10^8^ and 4 × 10^7^, respectively, the highest conjugation efficiency reached 5.53 × 10^−6^ (Table 1). We therefore took this parameter in our following conjugation experiments.

**Table 1:** Effects of the donor (*E. coli* cells)-to-recipient (*P. alni* Shahu spores) ratio on transconjugation efficiency. At ratio, three independent conjugations were analyzed.

Number of donor cells	Number of recipient spores	Number of exconjugants
1.0 × 10^9^	1.0 × 10^9^	(2.51 ± 0.17) × 10^−9^
	1.0 × 10^8^	(4.70 ± 0.33) × 10^−7^
	1.0 × 10^7^	(7.33 ± 0.87) × 10^−6^
	1.0 × 10^6^	(4.12 ± 0.54) × 10^−6^
1.0 × 10^8^	1.0 × 10^9^	(3.76 ± 0.35) × 10^−9^
	1.0 × 10^8^	(1.22 ± 0.22) × 10^−6^
	1.0 × 10^7^	(5.53 ± 0.27) × 10^−6^
	1.0 × 10^6^	(3.92 ± 0.66) × 10^−6^
1.0 × 10^7^	1.0 × 10^9^	(8.81 ± 0.98) × 10^−9^
	1.0 × 10^8^	(7.75 ± 0.32) × 10^−7^
	1.0 × 10^7^	(8.63 ± 0.23) × 10^−7^
	1.0 × 10^6^	(2.57 ± 0.56) × 10^−6^

### Chromosomal Integration of an Exogenous Gene Using the *Φ*C31 *att/int* System

**Figure 2:**
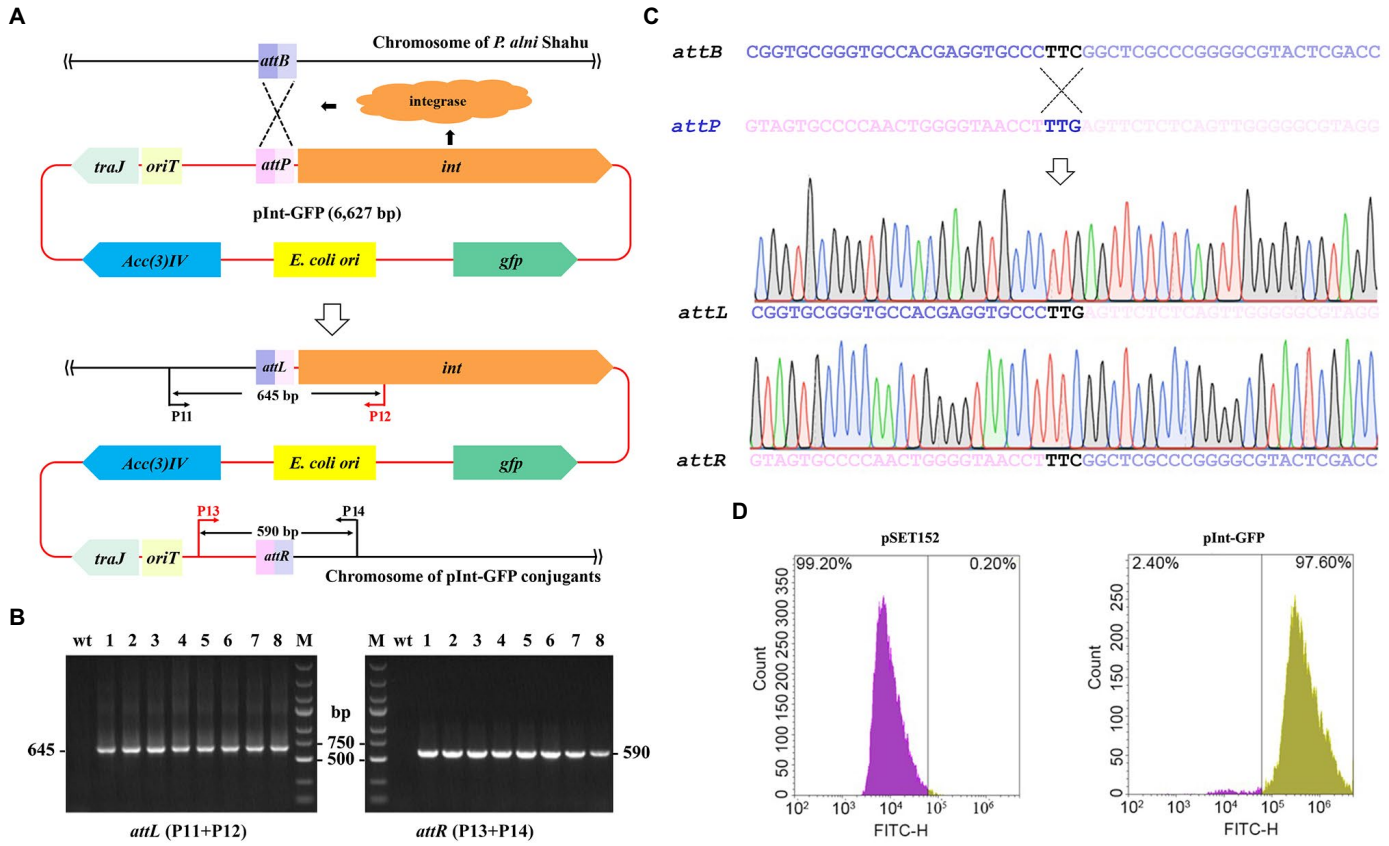
Chromosomal integration of the exogenous *gfp* gene using the *Φ*C31 *att/int* system. **(A)** Schematic showing integration of the entire pInt-green fluorescent protein (GFP) plasmid into *P. anli* Shahu chromosome. **(B)** Colony PCR analyses of eight randomly taken exconjugants, and the wild-type strain (wt) as a reference, using primer sets of P11 + P12 and P13 + P14 as shown in **(A)**. Predicted sizes of PCR products encompassing the created hybrid *attL* and *attR* sites after integration are indicated. M, DNA size marker. **(C)** Representative chromatographs of Sanger sequencing results of the *attL* and *attR* sites. Original sequences of *attB* in *P. anli* Shahu chromosome and *attP* in pInt-GFP plasmid, respectively, are shown. **(D)** Signal of GFP was detected in the pInt-GFP exconjugant whereas not in the pSET152 exconjugant by flow cytometry.

The apramycin-resistance ability of the obtained exconjugants is suggestive of the chromosomal integration of the pSET152 plasmid harboring the *aac(3)IV* apramycin resistance marker gene driven by the *Φ*C31 integrase (Figure 2A). Therein, integration of exogeneous genes into the *P. alni* Shahu chromosome could be achievable *via* the *Φ*C31 *att*/*int* system. To test such capability of the system, an integrative plasmid, pInt-GFP, was constructed by inserting a GFP-expressing cassette to pSET152 (Supplementary Figure S1). Then, conjugal transfer of pInt-GFP from *E. coli* to *P. alni* was conducted and its subsequent integration into the *P. alni* chromosome was examined.

According to the determined optimal ratio, *E. coli* ET12567 (pUZ8002, pInt-GFP) cells were mixed with *P. alni* Shahu spores for conjugation. Of the conjugants appeared on the plate with apramycin, eight were randomly picked up and grown up in a liquid ISP4 medium containing 50 mg/L of apramycin. To illustrate the chromosomal integration of pInt-GFP in detail, total DNAs were individually extracted from the selected exconjugants and used as templates for PCR analyses with a primer set of either P11 + P12 or P13 + P14 amplifying DNA fragments encompassing the *attL* or *attR* site, respectively (Figure 2A). Since P11 and P14 target the genomic sequences while P12 and P13 are located on the plasmid, PCR products can be amplified only when chromosomal integration of plasmid occurs. Agarose gel electrophoresis of the PCR products suggested successful integration of pInt-GFP into the Shahu genome, as PCR products with the expected sizes were amplified from the chromosomal DNAs of the conjugants but not from that of the wild-type strain (wt; Figure 2B). Subsequent Sanger sequencing of the PCR products confirmed the generation of the hybrid sequences of *attL* and *attR* from *attB* and *attP* (Figure 2C). In addition, our results detected the fluorescence signal of GFP from all the pInt-GFP exconjugants by flow cytometry, suggesting that the heterologously integrated GFP protein was stably expressed. In contrast, the signal was not detectable in the reference pSET152 exconjugant (Figure 2D). All these combined results suggested that the *Φ*C31 *att*/*int* system could be used for efficient chromosomal incorporation of foreign DNAs in *P. alni* Shahu.

### Generation of Gene Deletion Mutants of *P. alni* Shahu *via* Homologous Recombination

**Figure 3:**
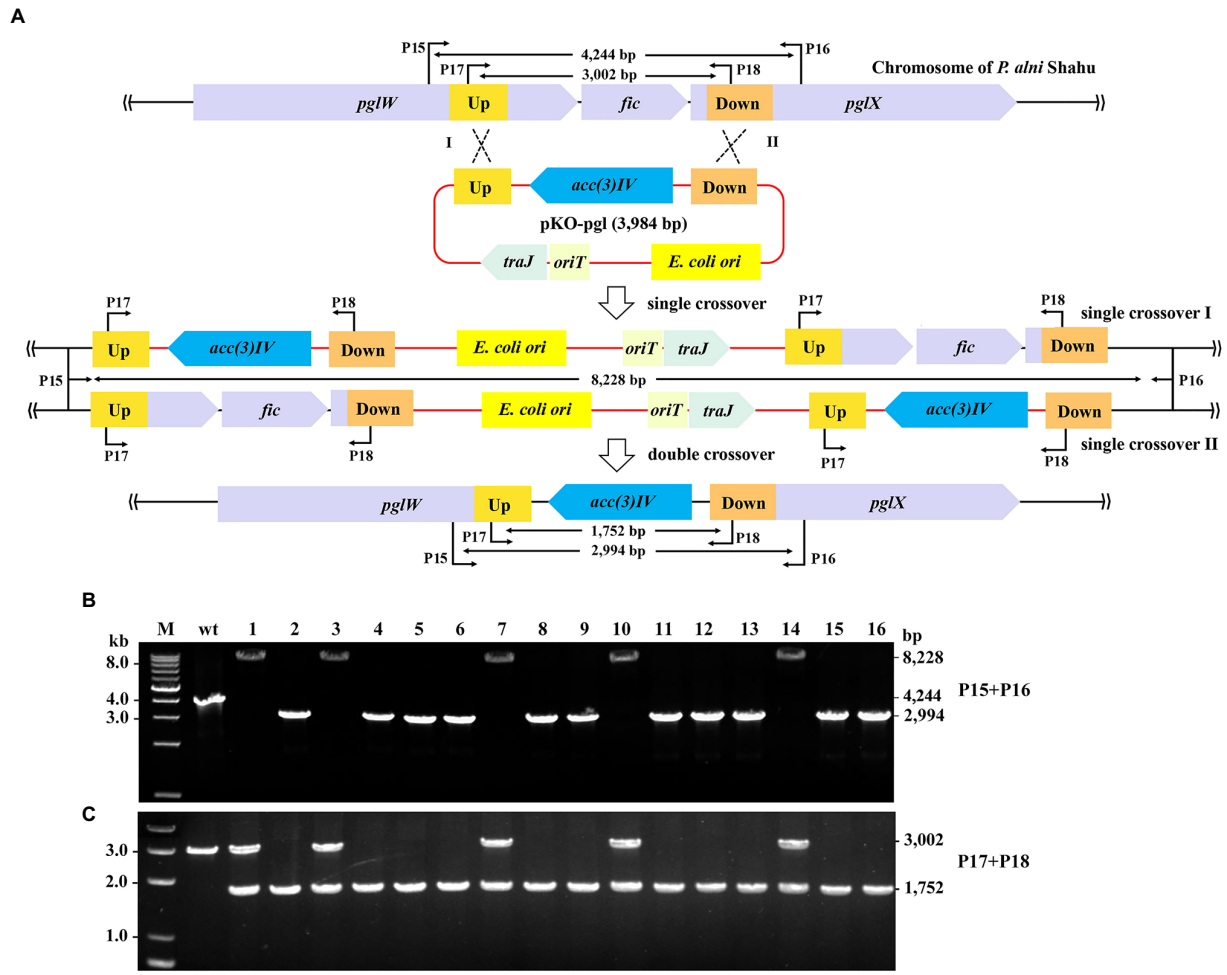
Gene deletion in *P. alni* Shahu *via* homologous recombination. **(A)** Scheme of deletion of the *pgl* genes using a suicidal non-replicating plasmid, pKO-pgl. Recombination between either the homologous upstream (Up) arms (Single crossover I), or downstream (Down) arms (Single crossover II), would result in integration of the entire plasmid into to the chromosome; while double crossover recombination was expected to occur, eventually leading to replacement of the target genes with the apramycin resistance marker, *acc(3)IV*. **(B,C)** PCR screening of *pgl*::*acc(3)IV* recombinants using primer sets of P15 + P16 **(B)** and P17 + 18 **(C)**. Predicted sizes of PCR products in wild-type (wt) and recombinants of single or double crossover recombination are indicated. M, DNA size marker.

We next demonstrated removal of exogeneous genes from the *P. alni* Shahu chromosome by using a non-replicating vector to facilitate homologous recombination. A DNA stretch containing the *pgl* genes was chosen as an editing target, since it was reported to be non-essential for cell viability (Goldfarb et al., 2015). The entire sequence of the *Φ*C31 *int* gene, together with that of the *attP* site, was removed from pSET152; while homologous arms of 633 and 733 bp were inserted upstream and downstream, respectively, of the *acc(3)IV* gene, giving the suicidal plasmid, pKO-pgl, for knockout of the *pgl* genes (Figure 3A).

After conjugal transfer of pKO-PGL into *P. alni* Shahu, colonies were observed on the selective plate containing apramycin, indicative of integration of the *acc(3)IV* gene into the host chromosome. We picked up 16 of the appeared colonies and analyzed their genotypes by PCR and Sanger sequencing. Using the primer set of P15 + P16, DNA products were amplified from total DNAs extracted from the strains that were expected to contain mutant alleles with a predicted size of 2,994 bp. However, the 2,994-bp band was amplified in only 11 of the tested colonies, while, interestingly, a predicted 8,228 bp band was amplified in the remainder (Figure 3B). Reasonably, double crossover has resulted in the replacement of the target region (2,260 bp) with the expression cassette of the *acc(3)IV* gene (1,010 bp), allowing for amplification of the 2,994-bp fragments (vs. the wild-type 4,244 bp); whereas a single crossover through either of the homologous arms would occur, leading to chromosomal integration of the entire plasmid and hence explaining the detected 8,228-bp DNA products.

To further corroborate the above observations, we designed an additional primer set, P17 + P18 with each, respectively, targeting a sequence located within the homologous arms, for PCR analyses of the same samples. As expected, PCR products of 3,002 and 1,752-bp were yielded in the wild-type cells and the double crossover recombinants, respectively; while both fragments were amplified in the colonies where single crossovers occurred (Figure 3C). These results thus confirmed the homologous recombination events deduced and showcased in Figure 3A.

## Discussion

Although successful examples of genetic engineering of some model actinobacteria have been accumulated in the literature (Deng et al., 2017; Kormanec et al., 2019), it is, in general, still more difficult to generically manipulate many rare actinomycetes. Methods to genetically manipulate the genome of rare actinomycetes, for example *Pseudonocardia*, might open new avenues toward the demonstration, development, and application of the valuable bacteria. Despite of the importance, until this work, there had been no detailed description of establishing a genetic manipulation system for genome modifications in any *Pseudonocardia* species.

The major challenges could include the transfer of the vector harboring the foreign DNAs into the host and the incorporation of DNA into the host chromosome. Intergeneric conjugation has been proven to be one of the most efficient approaches for gene transfer into actinomycetes, and has been already successfully applied to *Streptomyces* species as an example (Netzker et al., 2016; Kormanec et al., 2019). However, it was suggested that conjugation protocols have to be customized for individual species before accomplishing successful conjugation (Marcone et al., 2010), and indeed, our work has confirmed that the standard conjugation protocol (Rebets et al., 2017) is not suitable for *P. alni* Shahu. We figured out the optimal parameters for conjugation and successfully achieved transfer of the pSET152 plasmid and its derivatives from *E. coli* into *P. alni*, therein, making it possible to deliver the powerful CRISPR-Cas machinery and others of interest, into this bacterium by this very approach for high-throughput genome engineering in the future.

We did observe the integration of the transferred pInt-GFP, a pSET152 derivative, into the genome at an *attB* site. Based on the confirmed *attL* and *attR* sequences, we deduced the 51-nt sequence of the *attB* site (Figure 2C) and traced it back to the genome of *P. alni* Shahu, seeing that it laid within an OFR coding for a pirin-homolog. This was very similarly to the case of *Streptomyces* species where *attB* sites were exclusively located within pirin-encoding ORFs. We found that this *attB* sequence showed an 89% nt identity to the canonical *attB* sequence possessed by *Streptomyces coelicolor* A3(2) from which the antinophage *Φ*C31 was originated (Lomovskaya et al., 1972). The sequence identity between the *attB* site of *S. coelicolor* A3(2) and that of other actinomycetes seemed to play an important role in determining conjugation efficacy (Supplementary Figure S2). For example, plasmid transconjugation into *S. ambofaciens* harboring an *attB* sequence identical to that of *S. coelicolor* A3(2) (100% identity) yielded an efficiency of 1.4 × 10^−2^, while into *S. clavligerus* yielded an efficiency of 3 × 10^−5^ as the *attB* identity was 96% (Kim et al., 2008). Furthermore, when the plasmid was transconjugated into a non-*Streptomyces* species *Kitasatospora setae* with a 78% *attB* identity, the efficiency was dropped to 2 × 10^−7^ (Choi et al., 2004). This thus explained the observed efficiency of foreign DNA integration into *P. alni* Shahu genome (5.5 × 10^−6^, Table 1) *via* the *Φ*C31 *int/att* system in our work, and might provide a possibility to enhance the conjugation efficiency *via* engineering the *attB* sequence of *P. alni* Shahu to make it as identical as that of *S. coelicolor* A3(2).

An obvious shortcoming of this genetic manipulation system could be the lack of a counterselection marker. Currently, several suicide genes, e.g., *sacB*, *glkA*, *pyrF*, and *rpsL*, have been available as counterselection markers in different bacteria. The *sacB* gene, conferring sucrose sensitivity, was ever used for unmarked gene deletion in actinobacteria species (van der Geize et al., 2001, 2007). However, it was reported that this method was not applicable for *S. lividans* due to lack of sucrose sensitivity of this actinobacterium (Jager et al., 1992). The *glkA*, *pyrF*, and *rpsL*, and others, did play their roles in counterselection but only in the corresponding null mutants (van Wezel and Bibb, 1996; Hosted and Baltz, 1997; Knipfer et al., 1997). Additionally, some toxic genes, such as *mazF* (Zhang et al., 2006), can also be used as suicidal markers, which, however, rely on tightly controlled inducible promoters, thus limiting their applicability in strains with poorly developed molecular toolkits. Other methods may also include the application of temperature-sensitive replicons. For instance, the replicon of pIJ101 has been widely applied in some *Streptomyces* species (Mo et al., 2019). These methods might be also possibly applicable in *P. alni* Shahu, albeit, of course, necessitating the examination and determination of optimal parameters.

It would be also interesting to introduce the advanced CRISPR-Cas-based technologies into this actinobacterium. One of advantages of the technologies is that no selection marker is required due to the potent killing effect of the CRISPR-Cas systems on the bacterial cells. However, we could not get any transconjugant in the conjugation using a plasmid carrying a Cas9-expression cassette, even without a targeting guide RNA (data not shown). Possibly, the Cas9 nuclease *per se* is toxic to the host cells. Similar observations have been reported for other bacteria (Zheng et al., 2020; Hao et al., 2022). Interesting, we found that the chromosome of *P. alni* Shahu encodes a native CRISPR-Cas system. We believe that in the near future this system would be harnessed as a powerful genetic manipulation tool, although thorough demonstration of this system would be essentially needed.

Nevertheless, this work has enabled the generation of stable genome modifications in a rare actinomycetes *P. alni* Shahu with a considerably high efficiency, and thus making this strain amenable to the discovery and design of novel bioactive natural products by genetic engineering.

## Data Availability Statement

The datasets presented in this study can be found in online repositories. The names of the repository/repositories and accession number(s) can be found at: https://www.ncbi.nlm.nih.gov/, PRJNA563468; https://www.ncbi.nlm.nih.gov/genbank/, MW405797.

## Author Contributions

WP and YZe designed the research. JL, BW, QY, HS, and YZa performed the experiments. WP, JL, BW, and YZe wrote the manuscript. All authors contributed to data analyses, read, revised, and approved the final manuscript.

## Funding

This work was supported by the Open Funding Project of the State Key Laboratory of Biocatalysis and Enzyme Engineering (SKLBEE2021019), and the Innovation Base for Introducing Talents of Discipline of Hubei Province (2019BJH021). YZe and WP acknowledge the support from the State Key Laboratory of Biocatalysis and Enzyme Engineering.

## Conflict of Interest

The authors declare that the research was conducted in the absence of any commercial or financial relationships that could be construed as a potential conflict of interest.

## Publisher’s Note

All claims expressed in this article are solely those of the authors and do not necessarily represent those of their affiliated organizations, or those of the publisher, the editors and the reviewers. Any product that may be evaluated in this article, or claim that may be made by its manufacturer, is not guaranteed or endorsed by the publisher.
