# Supplementary material for: Enabling Efficient Genetic Manipulations in a Rare Actinomycete Pseudonocardia alni Shahu
Source: Front Microbiol. 2022 Mar 3;13:848964. doi: 10.3389/fmicb.2022.848964 (PMC8928166; doi:10.3389/fmicb.2022.848964)
Supplement: Supplementary file 1 [file Data_Sheet_1.docx]

**SUPPLEMENTAL INFORMATION**

**Figure S1. Procedure of plasmid construction.** (**a**) the starter vector pSET152. (**b**) construction of the integrative plasmid pInt-GFP. (**c**) construction of the non-replicative knockout plasmid pKO-pgl. Restriction sites for cloning and primers for amplification of inserts are indicated.


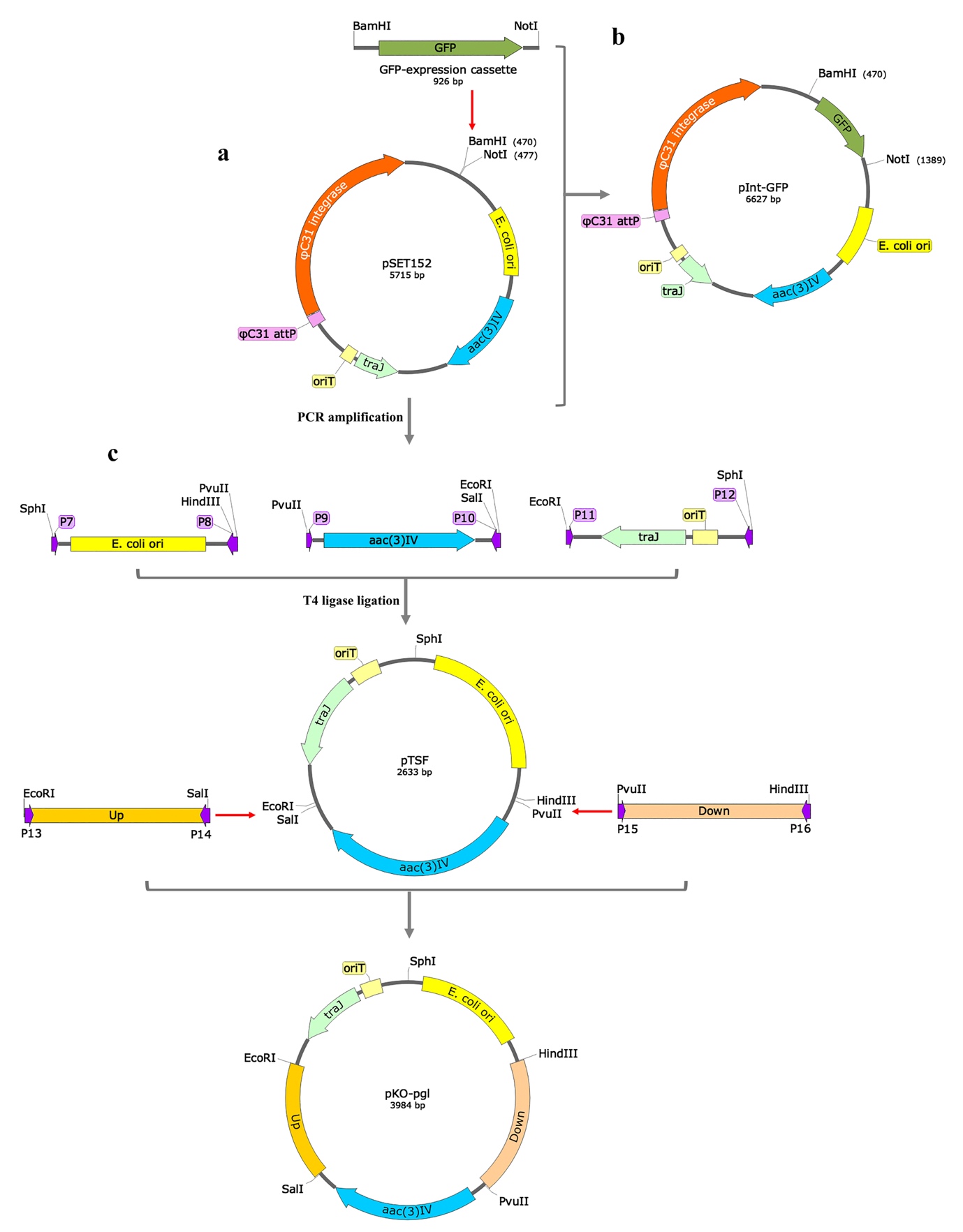


**Figure S2. Alignment of *attB* sequences.** Sequences of *attB* sites of *Pseudonocardia alni* Shahu (*Pal*), *Kitasatospora setae* (*Kse*) and some selected *Streptomyces* species, including *S. aureofaciens* (*Sau*), *S. cinnamonensis* (*Sci*), *S. griseus* ATCC 12475 (*Sgr*), *S. hygroscopicus* NRRL5491 (*Shy*), *S. longisporoflavus* 83E6 (*Slo*), *S. clavuligerus* (*Scl*), and *S. ambofaciens* ATCC15154 (*Sam*), were aligned with the canonical *attB* sequence of *S. coelicolor* A3(2) (*Sco*). Each site consists of a core motif (CCC/G) and two flanking sequences (*attB* and *attB’*)**.** The nucleotides of the canonical seqeuence were shaded with colours, *i.e.* C in blue, G in yellow, A in red, and T in green, while nucleotides in *attB* sites differing from the canonical sequence are shaded with the same respective colours.


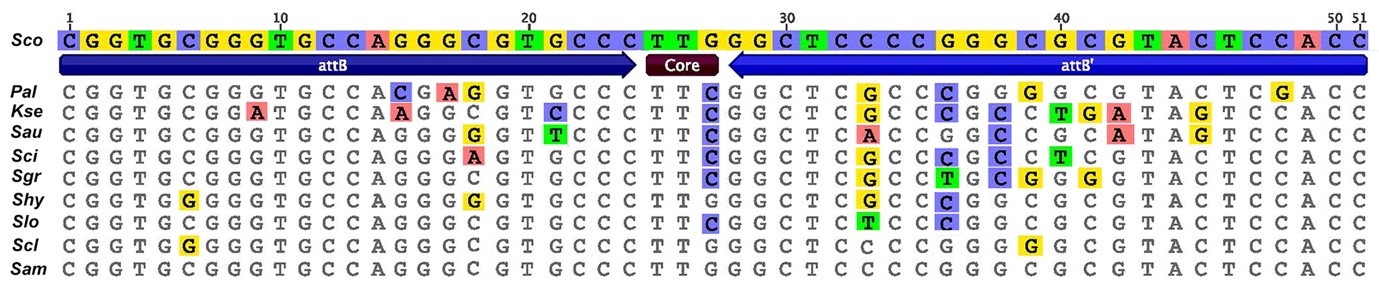


**Table S1**. Bacterial strains and plasmids used or constructed in this work.

| **Strains/Plasmids** | **Genotype and features** | **Source or reference** |
| --- | --- | --- |
| **Strains** |  |  |
| *Pseudonocardia alni* Shahu | Initial strain | This work |
| *E. coli* ET12567(pUZ8002) | *dam-13*::*Tn9*, *dcm-6*; pUZ8002^+^(Δ*oriT*); for conjugative transfer of DNAs | ([MacNeil et al., 1992](#_ENREF_16)) |
| *E. coli* DH5a | General cloning host strain | MBI Fermentas |
| *P. alni att*-GFP | Shahu derivative with the entire *ZMO0038* gene deleted | This work |
| *P. alni* D*pgl* | Shahu derivative with a DNA fragment in the *pgl* locus deleted | This work |
| **Plasmids** |  |  |
| pSET152 | Carrying *φ*C31 *attP* site and integrase gene (*int*), *traJ* and *oriT* of RK2, and *aac(3)IV* apramycin resistance gene | ([Flett et al., 1997](#_ENREF_7)) |
| pInt-GFP | pSET152 derivative; for site-specific chromosomal integration of *gfp* gene | This work |
| pTSF | A base vector consisting of three DNA fragments amplified from pSET152 through PCR; containing *φ*C31 *attP* site, the *traJ* and *oriT* of RK2, *aac(3)IV*, and the *E. coli ori* | This work |
| pKO-pgl | pTSF derivative containing a pair of recombination arms homologous to the sequences flanking the *pgl* target genes | This work |

**Table S2**. Oligonucleotides used in this work. Restriction sites underlined.

| **Oligonucleotide** | **Sequence (5’-3’)** |
| --- | --- |
| P1 | GCATGCAAGCTCTACTTCCTCGCTCACTGAC |
| P2 | AACCCAGCTGAACGGCAAAGCTTATGAACCAAAAGGATCTAGGTGAAG |
| P3 | GCTGTCAGCTGGTGCAGCTCCATCAGCAAAAGGGGAT |
| P4 | GCTGGAATTCCAGGGTTTGTCGACTATCATCAGCGAGCTGAAGAAAGAC |
| P5 | CCAGGAATTCTCGACCTGCAGGTCCCCGGGGATCG |
| P6 | GCACGCATGCCTCGACGGATCTTTTCCGCTGCATA |
| P7 | AGCAGAATTCGTCCTTGCGGCGCACCCCGGCCGCG |
| P8 | GCAGGTCGACGCGGTCGGCGTCTCCCAGCCGACCG |
| P9 | AACGCAGCTGAGCAGCCGGAACCGCGCGACCGACA |
| P10 | GGATAAGCTTCGGAACGCACCGCAGCCGGTTATCC |
| P11 | CGCGCAGCAGCGCGCCGCCGTCGGG |
| P12 | CATAGACAATGATCATGTTGAGCCG |
| P13 | AATCGCTATAATGACCCCGAAGCAG |
| P14 | CGTCATGGCGATCAGTCTGGCCCCC |
| P15 | TCTCGTCGAGATCACCGAGG |
| P16 | TCTCGATCAGCTTCTTCCCG |
| P17 | GCGGAACTGGCGACGACGGT |
| P18 | TCAGCGAGGTGCCGGAACGC |

**Table S3**. Information of the 16S rDNA of *P. alni* Shahu.

| **Accession number** | **Sequence** |
| --- | --- |
| MW405797 | GTTTGTTGCAAGTCAGGGTTTTGTTGGAGAGTTTGATCCTGGCTCAGGACGAACGCTGGC  GGCGTGCTTAACACATGCAAGTCGAGCGGTAAGGCCCTTTCGGGGGTACACGAGCGGCGA  ACGGGTGAGTAACACGTGGGTGACCTGCCCTCCACTCTGGGATAAGCCCGGGAAACTGGG  TCTAATACCGGATAGGACCACTGATCGCATGGTTGGTGGTGGAAAGTTTTTTCGGTGGGG  GATGGGCCCGCGGCCTATCAGCTTGTTGGTGGGGTGATGGCCTACCAAGGCGGTGACGGG  TAGCCGGCCTGAGAGGGCGACCGGCCACACTGGGACTGAGACACGGCCCAGACTCCTACG  GGAGGCAGCAGTGGGGAATATTGCGCAATGGGCGGAAGCCTGACGCAGCGACGCCGCGTG  GGGGATGACGGCCTTCGGGTTGTAAACCTCTTTCGCCAGGGACGAAGAGCGATTGACGGT  ACCTGGAGAAGAAGCACCGGCCAACTACGTGCCAGCAGCCGCGGTAACACGTAGGGTGCG  AGCGTTGTCCGGAATTATTGGGCGTAAAGAGCTCGTAGGCGGTGTGTCGCGTCGGCCGTG  AAAACTTGGGGCTTAACTCTGAGCGTGCGGTCGATACGGGCATCACTTGAGTTCGGCAGG  GGAGACTGGAATTCCTGGTGTAGCGGTGAAATGCGCAGATATCAGGAGGAACACCGGTGG  CGAAGGCGGGTCTCTGGGCCGATACTGACGCTGAGGAGCGAAAGCGTGGGGAGCGAACAG  GATTAGATACCCTGGTAGTCCACGCCGTAAACGTTGGGCGCTAGGTGTGGGGACCATTCC  ACGGTTTCTGTGCCGCAGCTAACGCATTAAGCGCCCCGCCTGGGGAGTACGGCCGCAAGG  CTAAAACTCAAAGGAATTGACGGGGGCCCGCACAAGCGGCGGAGCATGTGGATTAATTCG  ATGCAACGCGAAGAACCTTACCTGGGTTTGACATGCACCAGACATCCCTAGAGATAGGGC  TTCCCTTGTGGTTGGTGTGCAGGTGGTGCATGGCTGTCGTCAGCTCGTGTCGTGAGATGT  TGGGTTAAGTCCCGCAACGAGCGCAACCCTTGTTCCATGTTGCCAGCACGTAATGGTGGG  GACTCATGGGAGACTGCCGGGGTCAACTCGGAGGAAGGTGGGGATGACGTCAAGTCATCA  TGCCCCTTATGTCCAGGGCTTCACACATGCTACAATGGCTCATACAGAGGGCTGCGAGAC  CGTGAGGTGGAGCGAATCCCTTAAAGTGAGTCTCAGTTCGGATCGGGGTCTGCAACTCGA  CCCCGTGAAGTTGGAGTCGCTAGTAATCGCAGATCAGCAACGCTGCGGTGAATACGTTCC  CGGGCCTTGTACACACCGCCCGTCACGTCACGAAAGTTGGTAACACCCGAAGCCGGCGGC  CCAACCCTTGTGGAGGGAGCTGTCGAAGGTGGGACTGGCGATTGGGACGAAGTCGTAACA  AGGTAGCCGTACCGGAAGGTGCGGCTGGATCACCTCCTTT |
